# Developing and validating a survival prediction model based on blood exosomal ceRNA network in patients with PAAD

**DOI:** 10.1186/s12920-022-01409-3

**Published:** 2022-12-15

**Authors:** Shanshan Wang, Lijun Xu, Kangle Zhu, Huixia Zhu, Dan Zhang, Chongyu Wang, Qingqing Wang

**Affiliations:** 1 Department of General Surgery, Affiliated Hospital of Nantong University, Medical School of Nantong University, Nantong City, 226001 Jiangsu Province China; 2 Department of Medicine, Xinglin college, Nantong University, Nantong City, Jiangsu Province China; 3 Medical School of Nantong University, Nantong City, 226001 China

**Keywords:** Pancreatic adenocarcinoma, Exosomes, Competing endogenous RNA, Regulatory networks, Enrichment analysis, Survival prediction model

## Abstract

**Background:**

Among the most lethal cancers, pancreatic adenocarcinoma (PAAD) is an essential component of digestive system malignancies that still lacks effective diagnosis and treatment methods. As exosomes and competing endogenous RNA (ceRNA) regulatory networks in tumors go deeper, we expect to construct a ceRNA regulatory network derived from blood exosomes of PAAD patients by bioinformatics methods and develop a survival prediction model based on it.

**Methods:**

Blood exosome sequencing data of PAAD patients and normal controls were downloaded from the exoRbase database, and the expression profiles of exosomal mRNA, lncRNA, and circRNA were differentially analyzed by R. The related mRNA, circRNA, lncRNA, and their corresponding miRNA prediction data were imported into Cytoscape software to visualize the ceRNA network. Then, we conducted GO and KEGG enrichment analysis of mRNA in the ceRNA network. Genes that express differently in pancreatic cancer tissues compared with normal tissues and associate with survival (*P* < 0.05) were determined as Hub genes by GEPIA. We identified optimal prognosis-related differentially expressed mRNAs (DEmRNAs) and generated a risk score model by performing univariate and multivariate Cox regression analyses.

**Results:**

205 DEmRNAs, 118 differentially expressed lncRNAs (DElncRNAs), and 98 differentially expressed circRNAs (DEcircRNAs) were screened out. We constructed the ceRNA network, and a total of 26 mRNA nodes, 7 lncRNA nodes, 6 circRNA nodes, and 16 miRNA nodes were identified. KEGG enrichment analysis showed that the DEmRNAs in the regulatory network were mainly enriched in Human papillomavirus infection, PI3K-Akt signaling pathway, Osteoclast differentiation, and ECM-receptor interaction. Next, six hub genes (S100A14, KRT8, KRT19, MAL2, MYO5B, PSCA) were determined through GEPIA. They all showed significantly increased expression in cancer tissues compared with control groups, and their high expression pointed to adverse survival. Two optimal prognostic-related DEmRNAs, MYO5B (HR = 1.41, *P* < 0.05) and PSCA (HR = 1.10, *P* < 0.05) were included to construct the survival prediction model.

**Conclusion:**

In this study, we successfully constructed a ceRNA regulatory network in blood exosomes from PAAD patients and developed a two-gene survival prediction model that provided new targets which shall aid in diagnosing and treating PAAD.

## Introduction

Pancreatic adenocarcinoma (PAAD) is one of the most common malignant tumors in the digestive system. Given the latest cancer statistics in the US, the five-year survival rate of PAAD is less than 10% and ranks fourth among cancer-related deaths [1]. 53% of PAAD patients were already metastatic at diagnosis approximately, while the five-year survival rate in this group was only 2.4% [2]. Early detection and treatment are the only chance for patients to obtain radical treatment. However, due to the lack of typical clinical manifestations and specific tumor markers, the early screening of PAAD is strict. Carbohydrate antigen 19-9 (CA19-9) is currently the only serum biomarker for clinical use in pancreatic cancer management (an FDA-approved disease surveillance marker). However, neither CA19-9 sensitivity (mainly increased in advanced cancer) nor specificity (increased in non-PAAD and some benign diseases) is reliable in disease detection [3]. Therefore, it is of great significance to explore potential new targets for diagnosing and treating PAAD.

Non-coding RNAs, which include microRNAs (miRNAs) and long non-coding RNAs (lncRNAs), are involved in the regulatory processes of multiple biological information [4]. Researchers have recently found that non-coding RNAs are essential in regulating physiological function [5]. The ceRNA theory proposes that RNA could hold each other by competing for miRNA response elements [6]. Considerable studies have proved the role of the lncRNA-miRNA-mRNA regulatory network and that specific oncogenes have been identified in various cancers based on the ceRNA theory [7]. Therefore, the hypothesis of ceRNA regulatory networks and the research on the role of various ceRNAs in PAAD [8] could provide important clues and new research directions for the mechanism of tumor occurrence and development.

Exosomes are nano-sized (30–150 nm) extracellular vesicles released by a majority of distinctive types of cells and distributed in body fluid, such as cerebrospinal fluid, synovial fluid, saliva, cerebrospinal fluid, urine, breast milk, blood, and so on, carrying pathogenic miRNAs, lncRNAs, mRNAs, DNA fragments, and proteins [9, 10]. Exosomes transfer bioactive substances to receptor cells or activate signal transduction pathways in target cells to play the key function of intercellular communication, which has high clinical therapeutic and diagnostic value [11]. Research has validated that highly expressed exosomal CD44v6 and C1QBP are promising biomarkers to predict prognosis and liver metastasis in patients with PAAD [12]. However, ceRNA networks derived from exosomes have not been reported in PAAD.

This study aims to explore new targets for diagnosing and treating PAAD by constructing a survival prediction model based on a blood exosome-derived ceRNA network.

## Materials and methods

### Data download and screening of differentially expressed mRNA, lncRNA, and circRNA

All methods were carried out following relevant guidelines. The exoRBase (http://www.exoRBase.org) [13] is an online database encompassing mRNA, lncRNA, and circRNA extracted from RNA-seq investigations of human blood exosomes. First, we download the blood exosome sequencing data of PAAD patients and normal controls from exoRbase database 2.0 (http://www.exorbase.org/) with the corresponding gene annotation files downloaded. The gene expression profile matrix was integrated, and the gene annotation file was used to annotate the circRNA. The blood exosomes of PAAD patients were used as the experimental group (n = 164), and normal human blood exosomes as the control (n = 118). The differential expression profiles of mRNA, lncRNA and circRNA in exosomes were analyzed, respectively. The screening condition for differential expression was | log2FC |> 1, and the corrected screening condition was *P*-value < 0.01.

### Prediction of interacting miRNA and construction of ceRNA network

Target Scan Human7.2 (https://www.targetscan.org/vert_72/) [14] and miRanda (http://www.microrna.org/microrna/home.do) [15] were used to predict DEmRNAs-bound miRNAs. Next, we used miRcode (http://www.mircode.org/) [16] and ENCORI (http://starbase.sysu.edu.cn/) [17] to predict miRNAs binding to DElncRNAs while miRNAs that bind to DEcircRNAs were indicated by StarBase (http://starbase.sysu.edu.cn/) database [18] and circbank (http://www.circbank.cn/) [19]. Finally, we took the intersection of the miRNAs obtained from these three sets and imported the related mRNA, circRNA, lncRNA, and their corresponding miRNA prediction data into Cytoscape (version 3.8.2) software to visualize the ceRNA network [20].

### Functional enrichment analysis of differentially expressed mRNA

We used the GO annotation of the genes from the R software package ‘org.Hs.eg.db’ (version 4.1.0) and the KEGG rest API (https://www.kegg.jp/kegg/rest/keggapi.html) to acquire the latest KEGG Pathway gene annotation as a background, and then mapped the genes to the background set for enrichment analysis using the R software package ‘clusterProfiler’ (version 4.1.0) to obtain the results of gene set enrichment. *P* value < 0.01 and false discovery rate (FDR) < 0.1 were considered statistically significant.

### Expression and survival analysis of the Hub genes

The Gene Expression Profiling Interactive Analysis database (GEPIA; http://gepia.cancer-pku.cn) [21] was used to compare the expression difference and overall survival (OS) of genes in PAAD patients from the ceRNA network obtained before. Genes whose differential expression was evident and whose survival curve *P* value < 0.05 were determined as the Hub genes.

### Construction and validation of the prognostic model

Robust prognostic genes were obtained using both univariate and multivariable Cox regression based on the expression profiles and clinical information of 179 patients in the TCGA-PAAD cohort. Based on the prognostic Hub gene expression and prognostic coefficients, we calculated the risk score for each patient in the TCGA-PAAD cohort using the following formula: ExpGene1 * Coef1 + ExpGene2 * Coef2 + ExpGene3 * Coef3… The normalized expression value for each feature gene was Exp, and the regression coefficient for this gene in the multiple Cox regression analysis was Coef. After that, we classified the PAAD patients in the TCGA training set into the low-risk and high-risk groups according to the optimal cut-off value of risk score calculated by the R software package ‘maxstat’ (version 4.1.0). The Kaplan–Meier analysis was used to examine overall patient survival in the low and high-risk categories. The ROC curves were used to assess the performance of the risk scoring model through the “survival ROC” R package (version 4.1.0). In total, we validated 213 patients with the entire clinical features in the ICGC cohort to validate this risk model.

### Statistical analysis

Perl (version strawberry-Perl-5.32.) programming language is used to sort data, and R language (version 4.1.0) is used for data analysis and drawing. The measurement data are expressed as mean ± standard deviation, and the statistical test adopts a t-test or analysis of variance. *P* value < 0.05 was statistically significant.

## Results

### Data download and difference analysis

**Fig. 1:**
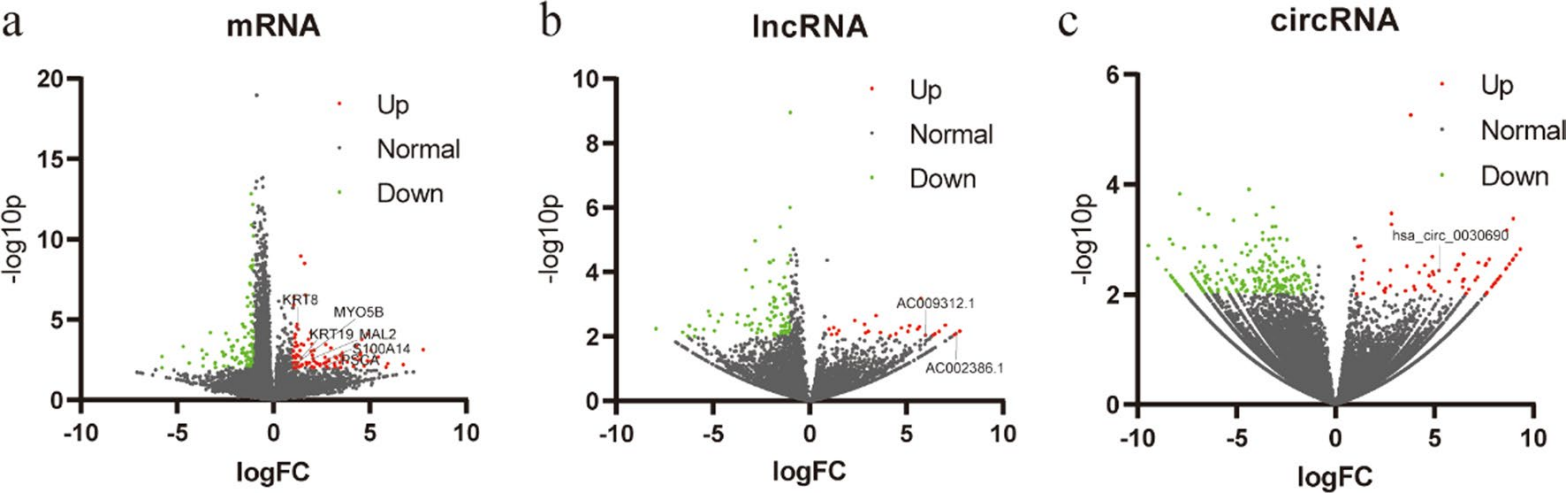
Volcano plots of differentially expressed profiles of exosomal RNAs between PAAD patients and normal controls. (**a**) mRNAs, (**b**) lncRNAs, (**c**) circRNAs. Red and green indicate up and downregulation, respectively

Sequencing data of the normal population (n = 118) and PAAD patients (n = 164) were downloaded from the exoRbase database. The mRNA expression profile, lncRNA expression profile, and circRNA expression profile matrix were integrated. The differences in mRNA, lncRNA, and circRNA were analyzed by network analysts, and the DEmRNAs (n = 205), DElncRNAs (n = 118), and DEcircRNAs (n = 98) were screened. Volcano maps (Fig. 1a–c) revealed the differential expression.

### Construction of miRNA-related ceRNA regulatory network

**Fig. 2:**
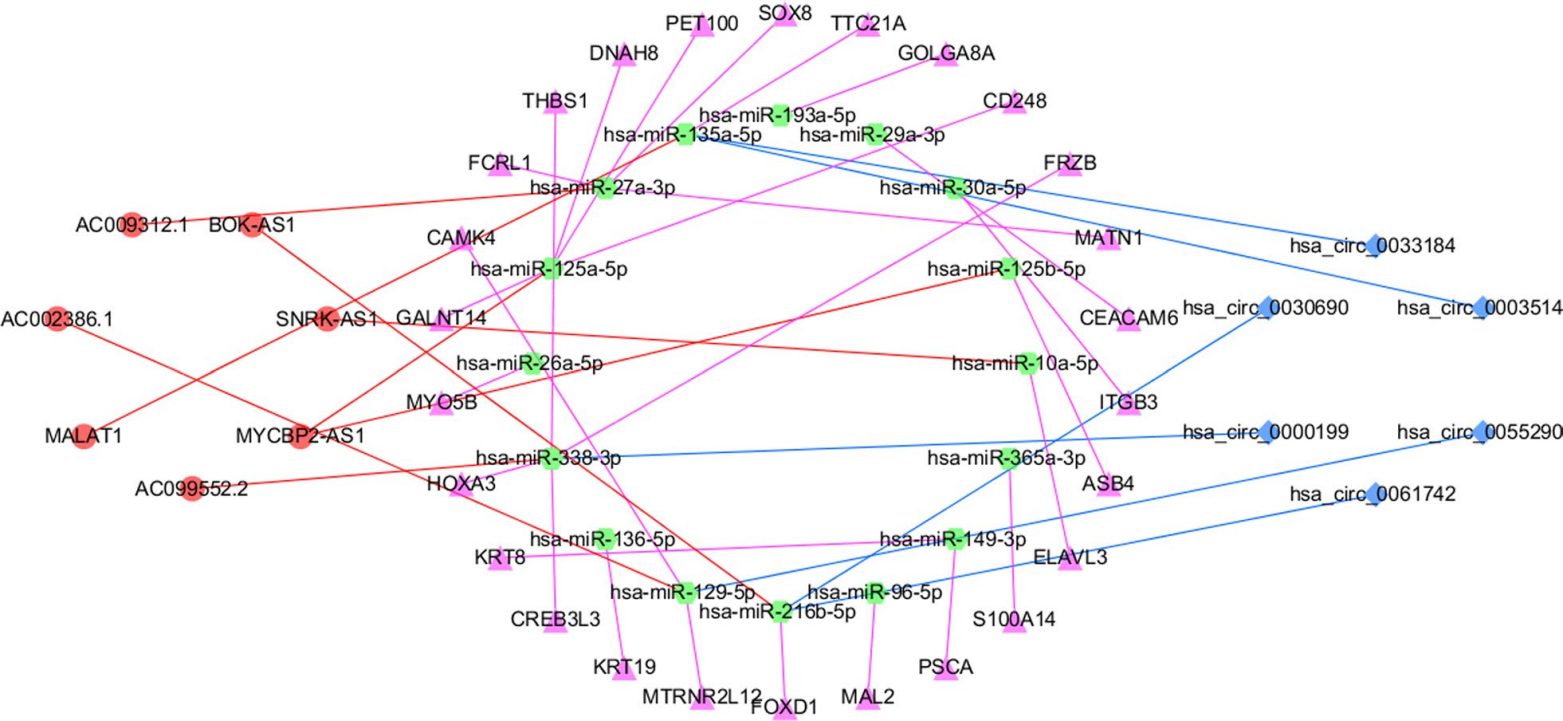
ceRNA network of differentially expressed genes. The red circle represents lncRNAs, the purple triangle represents mRNAs, the blue diamond represents circRNAs, and the green circle represents miRNAs

We used Targetscan Human7.2 and miRanda databases to predict the miRNA combined with DEmRNAs (n = 26), miRcode and ENCORI were used to predict the miRNA combined with DElncRNAs (n = 7), and Starbase and circbank database were used to indicate the miRNA combined with DEcircRNAs (n = 6). The ceRNA network was constructed by Cytoscape software, including 26 mRNA nodes, 7 lncRNA nodes, 6 circRNA nodes, and 16 miRNA nodes (Fig. 2).

### GO and KEGG pathway enrichment analysis

**Fig. 3:**
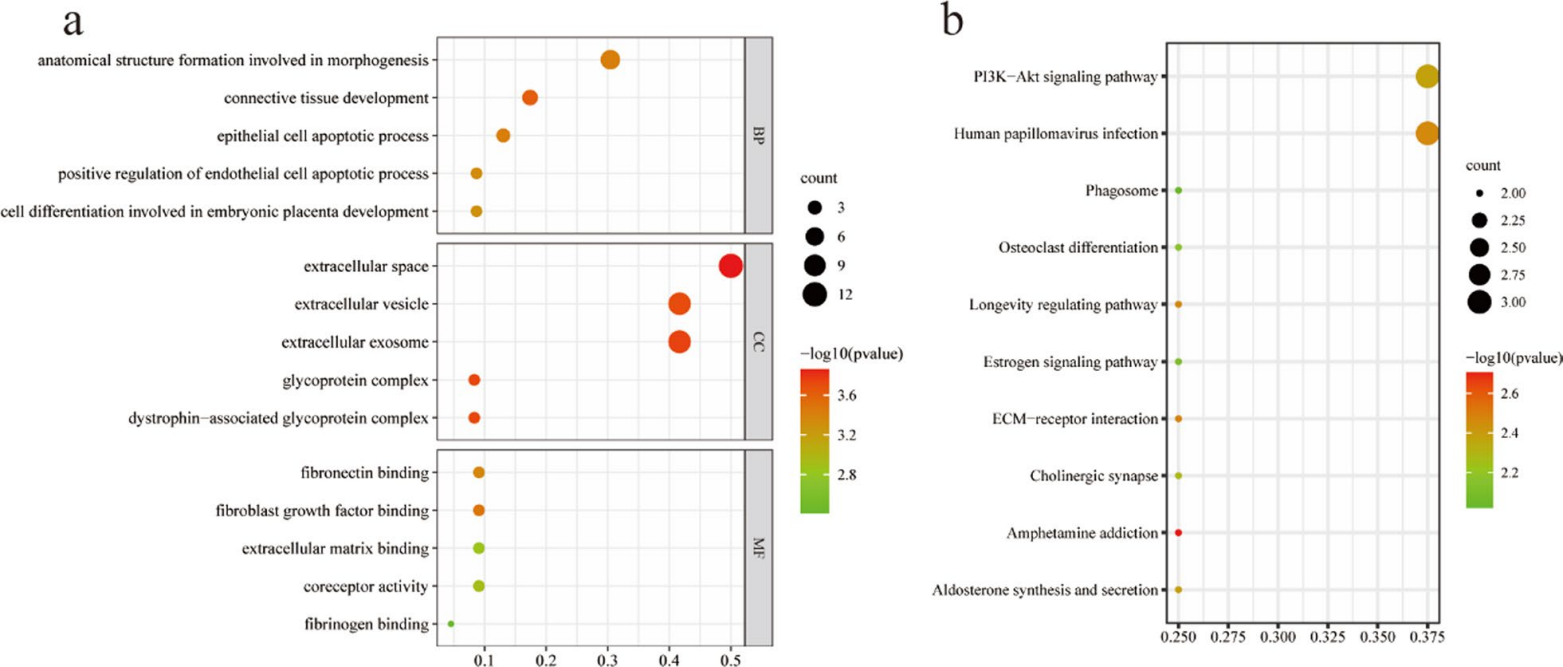
Bubble Diagram of GO and KEGG enrichment analysis of differential mRNAs. (**a**) BP, CC, and MF of the top 5 GO enrichment. (**b**) Top 10 KEGG enrichment. The gradient of green to red represents a change of *P* values from low and high, and the sizes of the dots represent related mRNA numbers. *BP* biological processes; *CC* cell components; *MF* molecular function

After the DEmRNAs in the constructed ceRNA network was converted from gene symbol to Entrez ID, GO and KEGG enrichment analysis was performed on the differential gene sets by R. GO annotation enrichment analysis showed that DEmRNAs were mainly enriched in anatomical structure formation involved in morphogenesis, connective tissue development, epithelial cell apoptotic process, positive regulation of endothelial cell apoptotic process, and cell differentiation involved in embryonic placenta development in biological processes (BP). Fibroblast growth factor binding, fibronectin binding, coreceptor activity, extracellular matrix binding, and fibrinogen binding were the most affected in molecular function (MF). The most abundant cell components (CC) were extracellular space, extracellular exosome, extracellular vesicle, dystrophin-associated glycoprotein complex, and glycoprotein complex (Fig. 3a). KEGG enrichment analysis showed that the DEmRNAs in the regulatory network were mainly enriched in Human papillomavirus infection, PI3K-Akt signaling pathway, Amphetamine addiction, ECM-receptor interaction, Longevity regulating pathway, Aldosterone synthesis, and secretion, Cholinergic synapse, Osteoclast differentiation, Estrogen signaling pathway, and Phagosome (Fig. 3b).

### Screening of the Hub genes

**Fig. 4:**
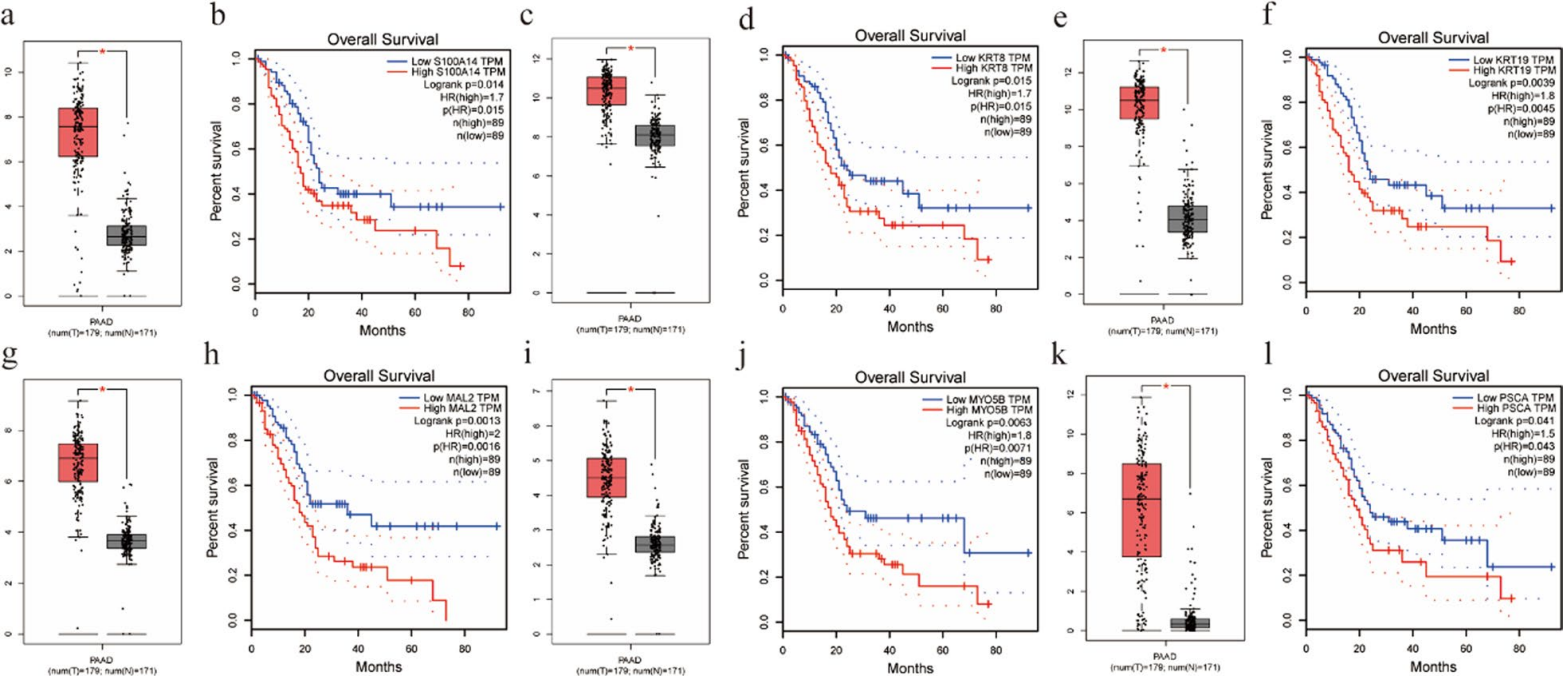
Expression boxplot and Kaplan–Meier survival curves of the DEmRNAs that are significantly associated with OS in PAAD patients. (**a**–**b**) S100A14, (**c**–**d**) KRT8, (**e**–**f**) KRT19, (**g**–**h**) MAL2, (**i**–**j**) MYO5B, (**k**–**l**) PSCA. *P < 0.05

We analyzed genes from the previous ceRNA network in GEPIA. We determined the genes that express differently in pancreatic cancer tissues compared with normal tissues and associate with survival (*P* < 0.05) as Hub genes, yielding a total of six Hub genes, including S100A14, KRT8, KRT19, MAL2, MYO5B, and PSCA (Fig. 4). These six genes were significantly highly expressed in pancreatic cancer tissues, and the high expression in pancreatic cancer patients was significantly associated with a poor prognosis.

### Construction and validation of the prognostic models

**Fig. 5:**
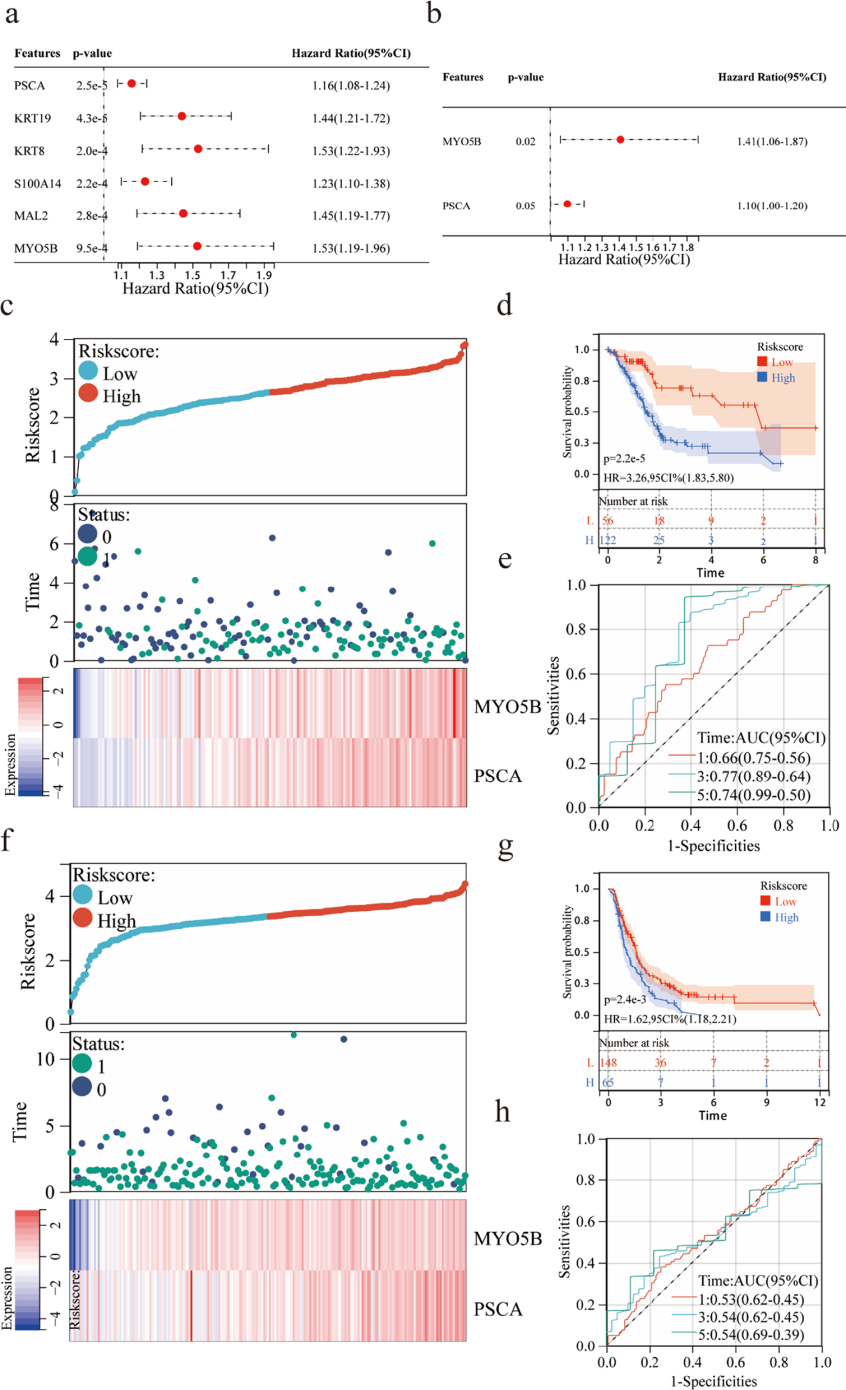
Construction and validation of the prognostic models by TCGA-PAAD dataset and ICGC dataset, respectively. (**a**–**b**) Univariate and Multivariate Cox regression analysis of six Hub genes with OS. (**c**) Risk score distribution and heatmap of the two genes in the model based on the TCGA-PAAD dataset. (**d**) Kaplan–Meier curve for the two-gene model based on the TCGA-PAAD dataset. (**e**) Time-dependent ROC analysis of the two-gene model for 1-, 3- and 5-year OS based on the TCGA-PAAD dataset. (**f**) Risk score distribution and heatmap of the two genes in the model based on the ICGC dataset. (**g**) Kaplan–Meier curve for the two-gene model based on the ICGC dataset. (**h**) Time-dependent ROC analysis of the two-gene model for 1-, 3- and 5-year OS based on the ICGC dataset

**Fig. 6:**
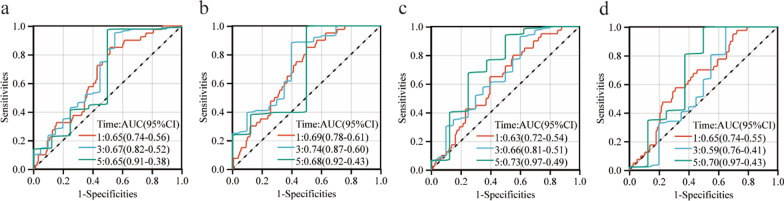
The time-dependent ROC analysis of the other four genes in univariate Cox regression analysis. (**a**) Time-dependent ROC analysis of KRT8 for 1-, 3- and 5-year OS. (**b**) Time-dependent ROC analysis of KRT19 for 1-, 3- and 5-year OS. (**c**) Time-dependent ROC analysis of MAL2 for 1-, 3- and 5-year OS. (**d**) Time-dependent ROC analysis of S100A14 for 1-, 3- and 5-year OS

We evaluated the prognostic value of the six Hub genes in the TCGA-PAAD cohort, assessed by both univariate and multivariate Cox regression analysis. PSCA and MYO5B were identified as prognostic biomarkers (Fig. 5a, b). Based on the expression levels and coefficients of the PSCA and MYO5B, we calculated the risk score for each patient as follows: Risk score = (0.1431) * PSCA + (0.4294) * MYO5B. The PAAD patients in the TCGA were classified into high-risk and low-risk groups based on the above formula. The survival state diagram clearly shows the survival state of each patient, and the heat map revealed the gene expression values of the model genes in each sample by color distribution (Fig. 5c). The Kaplan–Meier analysis demonstrated that the high-risk group had a significantly poorer OS compared with the low-risk group (hazard ratio [HR]: 3.26, 95% confidence interval [CI]: 1.83–5.80, *P* < 0.0001) (Fig. 5d). The area under the ROC curve (AUC) values showed that the risk score model performed well in predicting 1,3, and 5-year survival with AUC of 0.66, 0.77, and 0.74, respectively (Fig. 5e). In addition, we assessed the risk score model of 213 PAAD patients in the ICGC verification set and detected similar results (Fig. 5f–h). The overall survival time of the high-risk group was significantly lower than that of the low-risk group. The time-dependent ROC analysis showed that the AUC of the risk score model was 0.53 at 1-year, 0.54 at 3-years, and 0.54 at 5-years, indicating that it is reliable for predicting the survival of patients with PAAD. Although we did not include S100A14, KRT8, KRT19, and MAL2 in the prognostic model, the ROC curve showed that they also have predictive value for the survival of PAAD patients (Fig. 6a–d).

## Discussion

PAAD is one of the most common malignant tumors of the digestive tract. It has rapid progression, a low early detection rate, a poor prognosis, and an inferior survival rate [22, 23]. Early diagnosis and screening of PAAD is the best way to reduce its mortality rate and improve its prognosis, so early diagnosis of PAAD is a top priority.

With the understanding of exosomes going deeper, their roles in tumors achieve increasing interest [24, 25]. Reports showed that exosomes participate in the whole process of tumor development and development, mainly including the formation of the tumor microenvironment, tumor growth and metastasis, and the induction of the immune response [26]. Tumor cells can secrete more exosomes compared to normal cells, and exosomes provide a powerful diagnostic tool because their relative stability and composition cover all cancer-related biomarkers, including proteins, metabolites, DNA, DNA modifications, coding, and non-coding RNA, thus directly obtaining basic information about tumor cells [27, 28]. Our investigation constructed a ceRNA regulatory network in blood exosomes from PAAD patients and developed a two-gene survival prediction model, which shall be prospective biomarkers and therapeutic targets of PAAD. With the development of public databases, there are increasing methods for us to find effective biomarkers in various cancers [29]. This study utilized a complete exosome analysis via the exoRBase database on PAAD and normal blood samples; 205 DEmRNAs, 118 DElncRNAs, and 98 DEcircRNAs. Next, the ceRNA network was constructed by Cytoscape software, and a total of 26 mRNA nodes, 7 lncRNA nodes, 6 circRNA nodes, and 16 miRNA nodes were identified. Among them, TPT1-AS1 acts as an endogenous sponge of miR-30a-5p, increasing the levels of integrin β 3 (ITGB3) in pancreatic cancer cells [30]. Overexpression of miR-29a resulted in a significant decrease in the CEACAM6 protein levels, thereby inhibiting the migration and invasion of pancreatic cancer cells [31]. This ceRNA network narrowed the scope of research on potential candidate biomarkers for prognosis and treatment in PAAD.

The possible relevant functions of DEmRNAs in the ceRNA network were then investigated. In light of the KEGG analysis results, the DEmRNAs were abundant primarily in the Human papillomavirus infection, PI3K-Akt signaling pathway, Osteoclast differentiation, and ECM-receptor interaction. Among these pathways, the PI3K-Akt signaling pathway is widely involved in the development and development of pancreatic cancer [32, 33]. It is demonstrated that HOXA10-AS/miR-340-3p/HTR1D ceRNA axis promotes the adverse outcome of pancreatic cancer through the PI3K-AKT signaling pathway [34]. In addition, exosomes from pancreatic cancer cells have also been proven to induce osteoclast differentiation via the miR125a-5p/TNFRSF1B pathway [35]. Our findings on Human papillomavirus infection and ECM-receptor interaction are consistent with previous pancreatic cancer studies [36, 37].

Next, we screened the mRNAs in the ceRNA network by GEPIA to identify the genes highly expressed in pancreatic cancer tissues and associated with prognosis as Hub genes. S100A14, KRT8, KRT19, MAL2, MYO5B, and PSCA were determined as hub genes. S100A14 was significantly highly expressed in pancreatic cancer cells and tissues and promoted progression in pancreatic cancer [38]. KRT8 is composed of the main intermediate filament proteins expressed in single-layered epithelial cells of the gastrointestinal tract [39]. In gastric cancer, high KRT8 expression is illustrated to promote tumor progression and metastasis [40, 41]. Pistoni  et al. [42] also discovered overexpression of KRT8 in pancreatic cancer and its association with a high risk of developing pancreatic ductal adenocarcinoma. According to studies, KRT19 expression engages adverse tumor differentiation and aggressive behavior in hepatocellular carcinoma [43]. KRT19 is also proven to participate in immune evasion and is associated with poor prognosis in pancreatic cancer [44, 45]. Accumulating investigations pointed out that high MAL2 expression prompted pancreatic cancer progression and predicted adverse survival in patients with pancreatic cancer [46–48]. Notably, MYO5B was also up-regulated in pancreatic cancer and associated with poor prognosis [49]. PSCA is expressed in multiple cancers, and 60–80% of pancreatic tumors are documented to express PSCA in prior investigations [50]. Preclinical studies have demonstrated that targeting PSCA shall be a successful anti-tumor therapy for pancreatic cancer [50–53]. To screen genes showing a more significant advantage in pancreatic cancer prognosis, we then selected MYO5B and PSCA to establish a prognostic prediction model by univariate and multivariate cox regression analysis. Eventually, the risk-scoring model was also evaluated in the ICGC test verification set to ensure the accuracy and reliability of the prognostic prediction model, and similar results were detected. Collectively, the prediction model constructed above showed a significant advantage in pancreatic cancer and can be prognostic biomarkers and treatment targets.

There are still limitations in this study. First, the sample size is insufficient, and a validation set is required in the future. Second, the mechanisms by which the ceRNA network and Hub genes participate in the development of PAAD are unknown. In the future, further studies in cellular and animal experiments will be needed.

This study is the first to explore the differences in lncRNA, miRNA, and mRNA expression profiles to construct a ceRNA network in PAAD blood exosomes. Moreover, we developed and validated a survival prediction model based on this ceRNA network, which shall provide a potential target for the follow-up study of PAAD and even provide a potential biological marker for diagnosing and treating PAAD.

## Data Availability

The data that support the findings of this study are publicly available in the exoRbase repository at http://www.exorbase.org/.
